# Capturing intermediates and membrane remodeling in class III viral fusion

**DOI:** 10.1126/sciadv.adn8579

**Published:** 2024-12-04

**Authors:** Lenka Milojević, Zhu Si, Xian Xia, Lauren Chen, Yao He, Sijia Tang, Ming Luo, Z. Hong Zhou

**Affiliations:** ^1^ California NanoSystems Institute, University of California, Los Angeles, CA 90095, USA.; ^2^ Department of Chemistry and Biochemistry, University of California, Los Angeles, CA 90095, USA.; ^3^ Department of Microbiology, Immunology and Molecular Genetics, University of California, Los Angeles, CA 90095, USA.; ^4^ Department of Chemistry, Centre for Diagnostics and Therapeutics, Georgia State University, Atlanta, GA 30302, USA.; ^5^ Department of Chemistry, Institute of Biomedical Sciences, Georgia State University, Atlanta, GA 30302, USA.

## Abstract

Enveloped viruses enter cells by fusing their envelopes to host cell membranes. Vesicular stomatitis virus (VSV) glycoprotein (G) is a prototype for class III fusion proteins. Although structures of the stable pre- and postfusion ectodomain of G are known, its fusogenic intermediates are insufficiently characterized. Here, we incubated VSV virions with late endosome-mimicking liposomes at pH 5.5 and used cryo–electron tomography (cryo-ET) to visualize stages of VSV’s membrane fusion pathway, capture refolding intermediates of G, and reconstruct a sequence of G conformational changes. We observe that the G trimer disassembles into monomers and parallel dimers that explore a broad conformational space. Extended intermediates engage target membranes and mediate fusion, resulting in viral uncoating and linearization of the ribonucleoprotein genome. These viral fusion intermediates provide mechanistic insights into class III viral fusion processes, opening avenues for future research and structure-based design of fusion inhibition-based antiviral therapeutics.

## Teaser

Class III viral membrane fusion mechanisms of VSV captured by cryo-ET, providing mechanistic insights.

## INTRODUCTION

A critical step in host cell entry by an enveloped virus is fusion of the viral membrane with the host cellular membrane. This membrane fusion step is mediated by a surface glycoprotein (G) embedded in the viral envelope. Once an activating event triggers a conformational change within that fusion G, it assumes a putative extended intermediate state in which its hydrophobic fusion peptides/loops are positioned to be inserted in the target host membrane. Subsequent folding back of the extended intermediates leads to tight apposition and fusion of the two membranes via a hemifusion intermediate state. The bilayer fusion reaction typically follows a similar trajectory regardless of which class of fusion protein is catalyzing it (*1*–*4*).

**Fig. 1.**
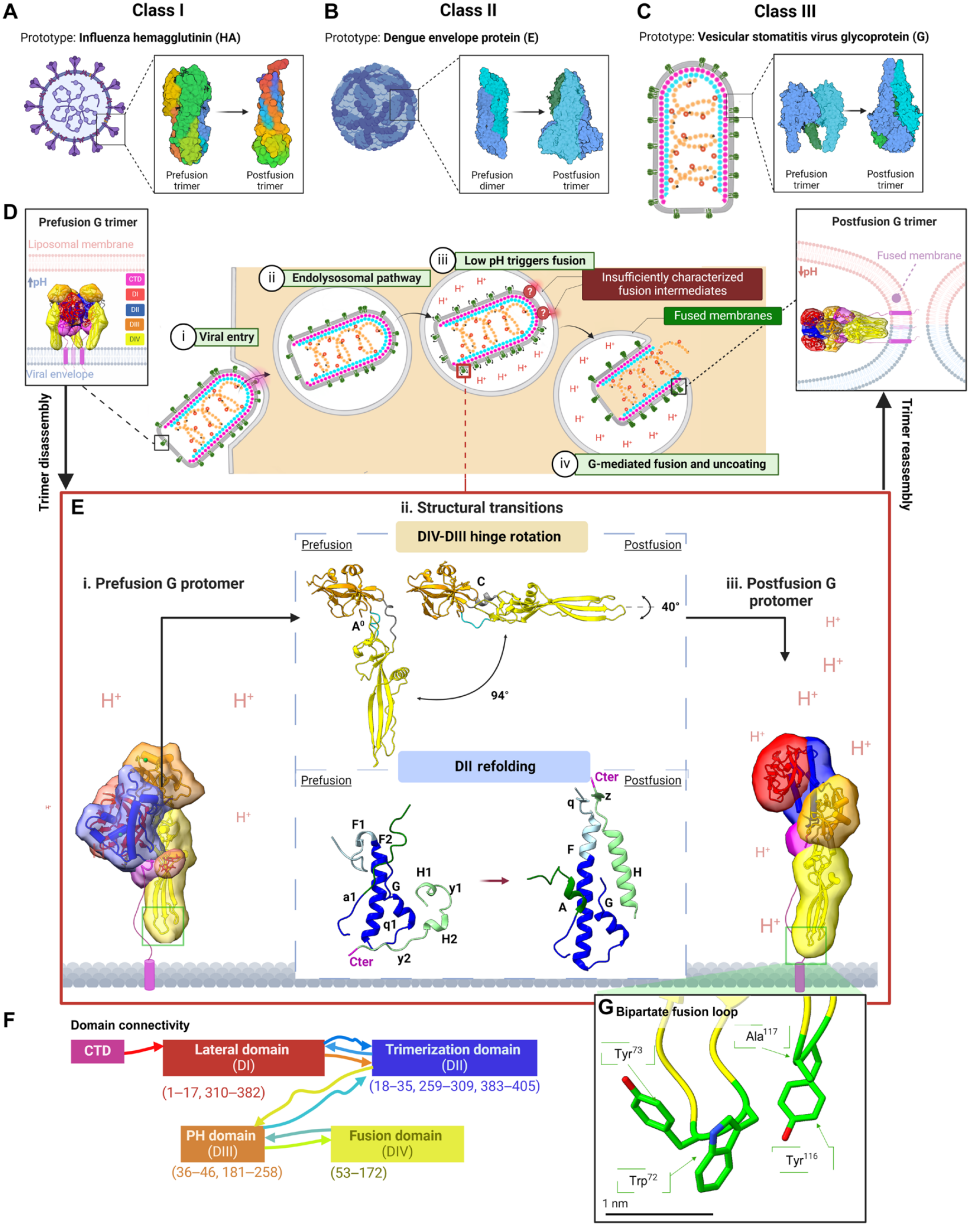
Overview of viral fusion protein classes and structural dynamics of class III fusion proteins. (**A** to **C**) Schematics of viruses bearing prototypical class I (A), II (B), and III (C) fusion proteins (left), alongside structures of pre- and postfusion trimeric conformations (right) of those proteins. (A) Class I represented with PDB entries 2YPG (*58*) for prefusion and 1QU1 for postfusion (*59*). (B) Class II represented with PDB entries 1OAN for prefusion (*60*) and 1OK8 for postfusion (*61*). (C) Class III represented with PDB entries 5I2S for prefusion (*15*) and 5I2M for postfusion (*9*). (**D** to **G**) Overview of the fusogenic structural transition and domain architecture of VSV G, the class III fusogen prototype. (D) Endocytosis of the VSV virion by a host cell is triggered by the binding of prefusion G trimers to LDL receptors on the host cell surface (i). The endosomal compartment engulfing the virion begins to acidify, leading to a fusogenic pre- to postfusion conformational shift within the trimer (iii–iv). Pre- (5I2S) and postfusion structures (5I2M) of G are displayed on the far left and far right sides of the figure, respectively, with their domains I–IV encased in 15 A masks and colored as annotated. (E) The prefusion G protomer (i) undergoes DIV-DIII hinge rotation and DII refolding (ii) en route to the postfusion conformation (iii). Black arrows connecting panels (D) and (E) highlight the premise that prefusion trimers disassemble during the fusogenic shift, and reassemble into postfusion trimers at the end. (F) Schematic illustrating the G protomer’s domain composition and connectivity, while (G) zooms into the structure of VSV’s bipartite fusion loop, which consists of four hydrophobic residues (green). The figure was created in BioRender.com.

Fusogenic viral Gs have been divided into three classes that are defined by their structure and fusion mechanism (*5*–*7*) (Fig. 1). The three classes were numbered (I to III) in the order in which they were discovered (Fig. 1, A to C), and this started in 1981 when the crystal structure of influenza hemagglutinin (HA) was solved (*8*). HA became the prototype for class I fusogens (Fig. 1A); within these proteins, a fusion peptide is located at the N-terminal region of the fusion subunit, followed by the N-terminal heptad repeat sequence 1 (HR1) and C-terminal heptad repeat sequence 2 (HR2) proceeding a transmembrane domain (TMD) at the C-terminal region (*5*). In the prefusion conformation of class I fusion proteins, the fusion peptide is buried in the trimeric interface. Along the entry pathway, a structural change of the trimeric G is triggered by a physical factor, such as decreasing pH, leading to the formation of an extended intermediate that may insert its N-terminal fusion peptides into the host membrane. In the postfusion conformation, refolding of HR2 on HR1 forms a six-helix bundle that brings the fusion peptides and TMDs together, as well as the two membranes, to complete the membrane fusion. In 1995, the envelope protein (E) of tick-borne encephalitis virus was resolved, defining a previously unidentified class of fusogens with marked structural differences from class I. In class II fusion Gs (Fig. 1B), the fusion peptide is a hydrophobic loop located in the middle of the fusion protein (*5*). A conformational change of the Gs dissociates prefusion dimers into monomers that can project their fusion loops into the host membrane. In the postfusion conformation, class II monomers are reorganized into trimers, which then mediate membrane fusion in a manner like that of class I fusion Gs. In 2006, the low-pH form of the vesicular stomatitis virus (VSV) G (*9*) and the herpes simplex virus–1 (HSV-1) G B (gB) (*10*) were both characterized by x-ray crystallography; these proteins from seemingly unrelated viruses were found to exhibit remarkable similarity to one another. They both appeared to be a hybrid of class I and class II fusion Gs and hence defined a third class of their own (Fig. 1C). The study of class III Gs in viruses like HSV-1 is complicated by the multitude of Gs on its surface (*11*, *12*). VSV G provides a more straightforward model for studying class III fusion because it is decorated by only one kind of G. This also makes VSV G protein a more favorable subject to structural investigation in situ.

The VSV G protein triggers clathrin-mediated endocytosis of the virion by binding members of the low-density lipoprotein–receptor family on the host cell surface (Fig. 1D, i and ii) (*13*, *14*). The endosomal environment triggers a low pH–induced structural transition within G that mediates fusion between the viral and endosomal membranes (Fig. 1D, iii and iv). Partial crystal structures of the G ectodomain have been determined previously as the neutral-pH prefusion and low-pH postfusion trimers (Fig. 1D, i and iv) (*9*, *15*). However, intermediate states on the transitional pathway from prefusion to postfusion remain inadequately characterized (Fig. 1Diii). G is divided into five domains (Fig. 1, E to G): a lateral domain (DI), a trimerization domain (DII), a pleckstrin homology domain (DIII), a fusion domain (DIV), and a C-terminal domain. The fusion domain of G protein has a four-residue bipartite fusion loop at its tip (Fig. 1Ef). Both the membrane-proximal C-terminal region and the TMD of G have been reported to play important roles in the fusion process (*16*–*20*). The endodomain of G protein is positioned to engage outer matrix (OM) proteins (*21*). Although this configuration is reminiscent of class II fusion proteins, the fusions loops of G protomers are not buried in the prefusion structure but rather point toward the viral membrane (*9*). The prefusion trimer interface is located at the trimerization domain, while its TMD anchors G in the viral envelope (*9*). In the postfusion conformation, segments connecting the domains reorganize to form a trimer that contains a six-helix bundle. The three fusion domains make trimeric contacts covered by the segments connecting the TMDs (*9*).

Structural comparison of the pre- and postfusion protomers have informed models for fusogenic conformational changes (*22*, *23*). Low pH is proposed to prompt relative movement of DIII and DIV domains around the bridging hinge (residues 47 to 52 and 173 to 180) and refolding of the DII-DIII connection (Fig. 1Eii). These shifts project the G protomer’s four-residue bipartite fusion loop (Fig. 1F), enabling its proximity to the target membranes. Low pH–triggered formation of this putative extended intermediate state is followed by refolding of the C-terminal domain and helix H, which results in the protomer’s collapse to a postfusion conformation (Fig. 1E, i and iii). However, the large differences in the trimer interfaces between the pre- and postfusion structures of G present a topological problem that necessitates first disassembly and then reassembly of the trimer during the pre- to postfusion transition. During this process, G trimers may be dissociated to monomeric protomers (*24*, *25*). It has also been suggested that a G antiparallel dimer may form as an intermediate (*26*), similarly to class II fusion Gs (*27*) that are present as antiparallel dimers in the prefusion conformation (Fig. 1B); over the course of the fusogenic transition, class II Gs are extended from the viral envelope and then reorganized into postfusion trimers (Fig. 1B) that are reminiscent of class III postfusion trimers (Fig. 1, D iv and Eiii). However, mechanistic insight into class III fusion is lacking compared to that of classes’ and requires further characterization (Fig. 1Diii).

Here, we use an integrated quantitative and structural approach to successfully observe two principal phenomena: first, the intermediate stages of bilayer fusion between VSV and liposomes (membrane apposition, hemifusion, the fusion pore, etc.), which are driven by G conformational changes; and second, the conformational intermediates of the G protein at endosomal pH (i.e., pH = 5.5). A range of intermediate structural states of G are captured using cryo–electron tomography (cryo-ET) and are categorized on the basis of morphology and spatiotemporal distribution. We observe that prefusion trimers disassemble into flexible and extended monomeric and dimeric intermediates that refold, mediating membrane remodeling and fusion that is followed by linearization of the viral genome. Our findings shed light on class III fusion processes and membrane remodeling events that have not been visualized in situ.

## RESULTS

### Stages of G-mediated fusion pore formation

To capture the dynamic interaction events between G on VSV virions and target membranes within the low-pH environment of late endosomes, we incubated purified VSV virions with liposomes at the endosomal pH (i.e., pH = 5.5) and performed cryo-ET in a Titan Krios instrument equipped with an energy filter and direct electron detector (fig. S1). Liposomes were prepared to mimic the composition of the late endosome (*28*–*30*), incorporating 1,2-dioleoyl-*sn*-glycero-3-phosphocholine (DOPC), 1,2-dioleoyl-*sn*-glycero-3-phosphatidylserine (DOPS), and cholesterol in a molar ratio of 4:2:1. The internal pH of liposomes (pH = 7.4) was intended to mimic cytosolic pH (pH = ~7 to 7.4). Mixtures of VSV and liposomes were incubated at pH 5.5 for 90, 120, and 150 s, yielding datasets containing 45, 13, and 42 tomograms, respectively (fig. S1, iii and iv). The choices of time points were based on empirical observations with the aim to capture intermediate states. The selected incubation times were optimized to enhance the probability of concurrently capturing membrane remodeling events and G conformational intermediates, enabling observation before the widespread fusion of virions with liposomes.

**Fig. 2.**
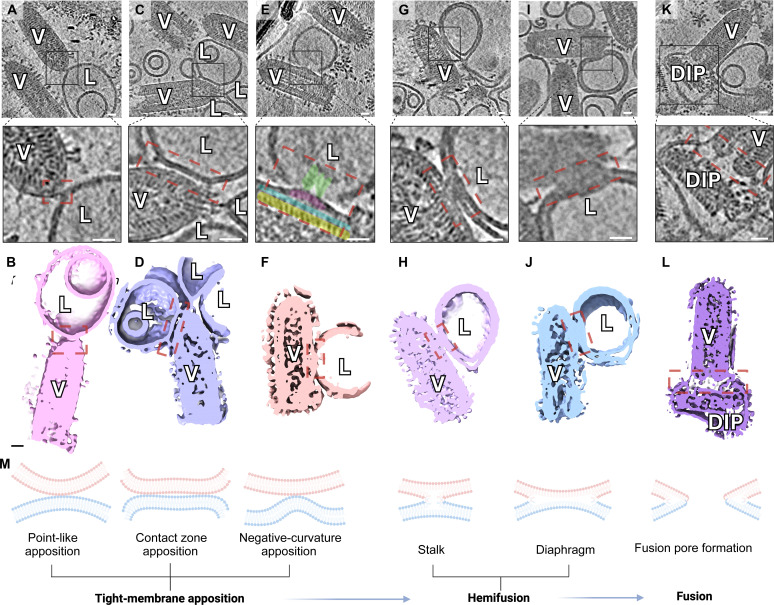
Visualizing membrane remodeling en route to fusion pore formation. Top row: Enlarged XY slice views of tomograms exemplifying instances of VSV-liposome complexes captured at fusion stages interpreted in (**M**). Second row: Below each of the top row panels is a corresponding zoomed inset of the contact zone, encased with black borders; contacts between membranes are enclosed by red dashed rectangles. Third row: Side cross views of 3D rendered features from their corresponding column. Fusion stages depicted and interpreted include point-like apposition (**A** and **B**), contact zone apposition (**C** and **D**), negative curvature apposition (**E** and **F**), the hemifusion stalk (**G** and **H**), the hemifusion diaphragm (**I** and **J**), and the fusion pore (**K** and **L**). Scale bars, 20 nm. The figure was created in BioRender.com.

While most virions in the population had not completed the membrane fusion process within 150 s, analysis of membrane fusion intermediates from 100 tomograms allowed us to reconstruct a sequence of events leading up to fusion pore formation (Fig. 2). Membrane recruitment and attachment by hyperextended G proteins (~17 to 19 nm) can be observed at each time point, although extended G intermediates (*n* = 371) are most prevalent at 150 s, making up 47.1% of all observed intermediates (shown below).

Once a liposome has been recruited and penetrated by extended G proteins, those proceed to fold back, causing tight superposition of opposing membranes. VSV interacted with liposomes in a variety of ways via its G proteins, and we attempted to qualitatively classify these interactions. From analysis of 327 VSV-liposome contact zones, we define three broad categories of tight membrane apposition (Fig. 2M) which we term point-like apposition (Fig. 2, A and B), contact zone apposition (Fig. 2, C and D), and negative curvature apposition (Fig. 2, E and F); apposition events do not involve, but precede, the fusion of proximal leaflets. Point-like apposition (Fig. 2, A and B) involves fold-back by a smaller number of fusogenic intermediates clustered around a narrow patch of membrane, causing a point-like protrusion in one membrane while the other remains flat (*31*); the protrusion is essential for reducing hydration repulsion between the polar head groups of lipidic membranes connected by surface water (*32*). On the other hand, we use the term “contact zone apposition” to describe superposition of a broad surface area, mediated by distal G proteins (Fig. 2, C and D). Contact zone apposition, involving a larger array of G protein intermediates, may overcome membrane resistance more effectively than the fewer intermediates in point-like apposition; this hypothesis appears likely considering reports that three to five G intermediates may be required to overcome membrane resistance, within a contact zone of 30 to 50 G intermediates (*23*).

Membrane dehydration, protein insertion, and increased lipid density could cause a bending moment in a bilayer to promote negative curvature and consequent curvature stress. In instances we refer to as negative curvature apposition (Fig. 2, E and F), application of sufficient force by fusogen fold-back can induce a ~50-nm-wide protrusion of the viral envelope, characterized by detachment of OM proteins from inner matrix (IM) and the nucleocapsid (Fig. 2C). Negative curvature was primarily observed during apposition and not during hemifusion. We interpret that negative curvature of the viral envelope is likely transient in the prehemifusion stage and quickly dissipates upon lipid mixing and removal of curvature stress.

Apposition leads to lipid mixing, starting with the formation of a minimal lipid connection by merger of the proximal leaflets of apposing bilayers, termed the fusion stalk (Fig. 2D). We observe that nucleation and expansion of the fusion stalk into a ~30- to 50-nm fusion diaphragm (Fig. 2E) is followed by formation of an enlarging fusion pore (Fig. 2F), which completes the fusion process.

### Ribonucleoprotein linearization after fusion with liposomes

**Fig. 3.**
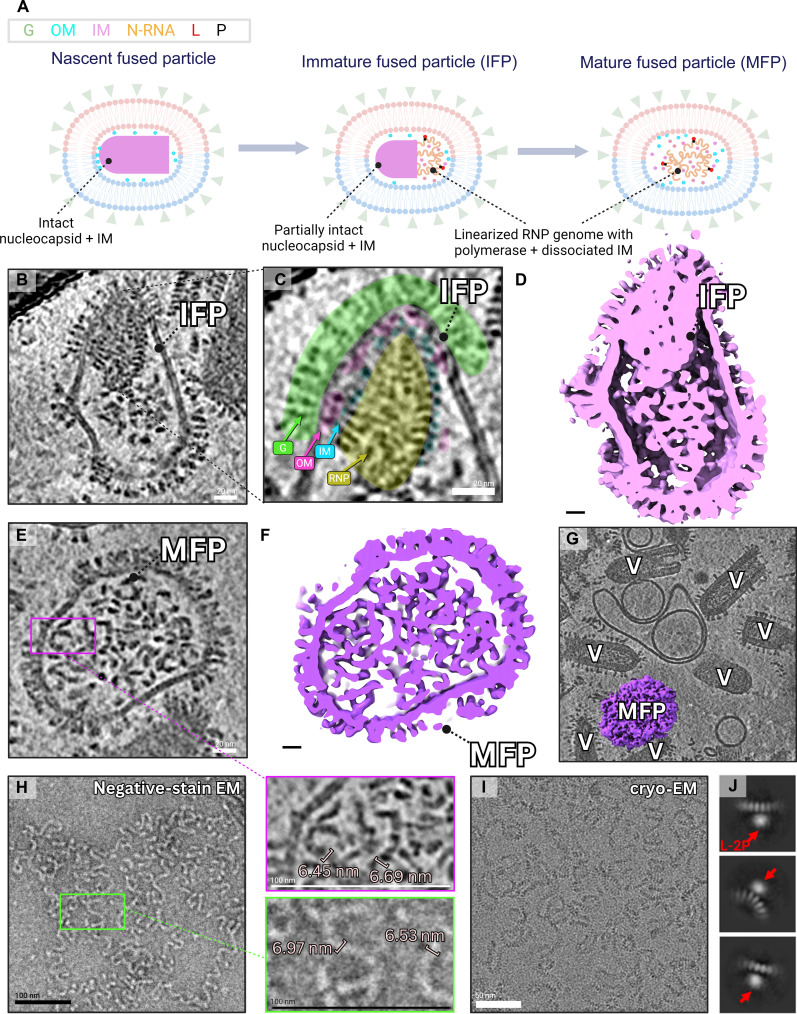
Maturation of the fused particle and visualization of the uncoated genome. (**A**) Cartoon representation of proposed postfusion alterations within the fused particle. (**B** to **D**) An enlarged XY slice view through a tomogram containing an immature fused particle (IFP) (B), along with a zoomed inset highlighting proteins within and surrounding the structured region of the nucleocapsid (C). The feature from (B) is 3D rendered and presented as a cross-sectional side view in (D). Scale bars, 20 nm. (**E** to **G**) Enlarged XY slice view through a tomogram containing a mature fused particle (MFP) (E) is supplemented with a zoomed inset (magenta), highlighting RNP thickness and strand morphology. (E) is 3D rendered and presented as a cross-sectional side view in (F). (G) XY slice view of the tomogram containing the MFP from (E) and (F), presented with the MFP’s corresponding density in purple. Scale bars, 20 nm. (**H**) Negative-stain TEM micrograph of a sample containing VSV RNP complexed with L-2P polymerase (left) along with a zoomed inset (green) to highlight morphology and strand thickness (right); the inset (green) is to scale with the inset from (E) (magenta), and the strand thickness is shown to be comparable. Scale bars, 100 nm. (**I** and **J**) Cryo-EM micrograph of the sample from (H) alongside select 2D classes demonstrating the presence of polymerase (red arrows) bound to the linearized RNP. Box size is 44 nm. Scale bar, 50 nm. The figure was created in BioRender.com.

From our observations (Fig. 3), we propose a model for the maturation of fused particles resulting from VSV uncoating into liposomes (internal pH = 7.4) at the external pH of 5.5 (Fig. 3A); this event models the release of the ribonucleoprotein (RNP) genome from within the endosome (pH = ~5.5) into the host cell cytosol (pH = ~7 to 7.4) after G-mediated fusion. We hypothesize that the fusion process may disrupt M protein binding, leading to linearization of the uncoated RNP genome with retained contacts to the viral polymerase; linearization of the RNP may facilitate polymerase motility during replication and transcription within the host cell cytosol.

In our system, nascent fused particles consist of an intact nucleocapsid with some portion of M proteins attached, encased in a fused envelope decorated with postfusion G trimers. Here, we refer to fused particles encasing partially intact nucleocapsids as immature fused particles (IFPs) (Fig. 3, A to D). However, most fused particles observed after 90 to 150 s were characterized by complete detachment of M proteins and linearization of the RNP genome (Fig. 3, A, E, and F), implying that this may be a rapid process. By analyzing the topology of fused particles at various stages of maturation, we can gather insights into the disassembly mechanisms of VSV structural proteins and viral uncoating.

OM proteins in IFPs are primarily observed adhering to IM in membrane-proximal regions, where OM are stabilized by the G endodomain (Fig. 3, B and C). This is in line with our previous observations of OM dissociating from IM and adhering to the interior face of the viral envelope in regions where negative curvature occurs (Fig. 2, C and D). We frequently observe that OM proteins dissociate from the rest of the virion in the event of any kind of virion disassembly and/or envelope drift; they appear to have an affinity for the G endodomain and remain membrane proximal primarily in regions below G trimers (Fig. 3C and fig. S2). Previous studies have found G trimers to associate with OM, with three endodomains from a G trimer reaching out to engage three OM sites spanning two turns and each OM site accommodating the endodomain of a neighboring G trimer (*21*). The endodomain of G contains as many as seven positively charged residues, which are expected to interact with a negatively charged helix (Val^189^-Tyr^192^) on the surface of OM.

IM demonstrates a more stable affinity for the nucleocapsid than to OM and is observed to be RNP bound even in the absence of OM (Fig. 3C and fig. S2, C and E). However, it does progressively shed from the nucleocapsid during postfusion events, which likely contributes to RNP linearization. IM has been proposed to maintain helical morphology of the nucleocapsid and its bullet shape (*21*); this is in line with our observation that completely bare helical RNPs always appear tube shaped, with no semblance of a domed tip in the absence of IM and OM proteins (fig. S3).

The mature fused particle (MFP; Fig. 3, E and F) contains linearized VSV RNP in complex with the viral polymerase and surrounded by free IM and OM proteins that had detached from the nucleocapsid. The size of MFPs varied from approximately the size of neighboring virions (Fig. 3G) to larger, and this is likely due to variance in the size of the liposome the virion had fused with. Fusion between G-decorated particles [virions, defective interfering particles (DIPs), and fused particles] was also observed (e.g., Fig. 2, K and L). Our interpretation of fused particle composition and RNP linearization (Fig. 3A) was supported by negative-stain transmission electron microscopy (TEM) (Fig. 3H) and cryo–electron microscopy (cryo-EM) single particle analysis (SPA) (Fig. 3I) of the VSV RNP in complex with polymerase at pH 8.0; after removal of the G and M proteins through detergent treatment of purified virions, the RNP chain exhibited equivalent topology as well as dimensions to density within MFPs (Fig. 3E). Two-dimensional (2D) classes from single-particle cryo-EM (Fig. 3J) using cryoSPARC (*33*) was used to confirm the presence of polymerases attached to the linearized RNP, which demonstrates that the transition from helical to linear topology does not disrupt L-2P binding. Furthermore, we captured helical RNPs mid-disassembly that also exhibited this topology in the linearized portion of their structures (figs. S2, B and D, and S3C). While there is a possibility that M proteins may be establishing other interactions after detachment from the nucleocapsid, we do not directly observe any proteinaceous aggregates or interactions distinct from the RNP because of limitations in resolution.

In summary, postfusion changes to VSV’s architecture involve breaking of most radial (IM-N, IM-OM, and OM-G) and vertical (IM-IM) interactions between VSV structural proteins, while lateral (N-N) interactions as well as RNA and L-2P contacts are preserved. Our observations suggest that (i) OM proteins have a higher affinity for the G endodomain than for IM proteins, which have a higher affinity for N proteins than for OM (Figs. 2C and 3C and fig. S2); (ii) uncoated RNP helices shed both layers of matrix protein before linearizing (Fig. 3C and figs. S2 and S3), and (iii) the viral polymerase remains bound to the RNP genome throughout the transition from helical to linear topology. Considering that RNPs are observed to be linearizing in a variety of microenvironments with a range of pHs [uncoated into liposomes (Fig. 3, B to E), breaking out of viral envelopes (fig. S2) as well as uncoated at pH 5.5 (fig. S3)], it is likely that this is a spontaneous process resulting from M protein detachment and independent of pH. While fusion-associated pH shifts or mechanical changes may be affecting the detachment of M proteins, our cryo-ET data offers limited insight to the chemistry underlining this process.

### Direct segmentation of G from missing wedge-corrected tomograms

**Fig. 4.**
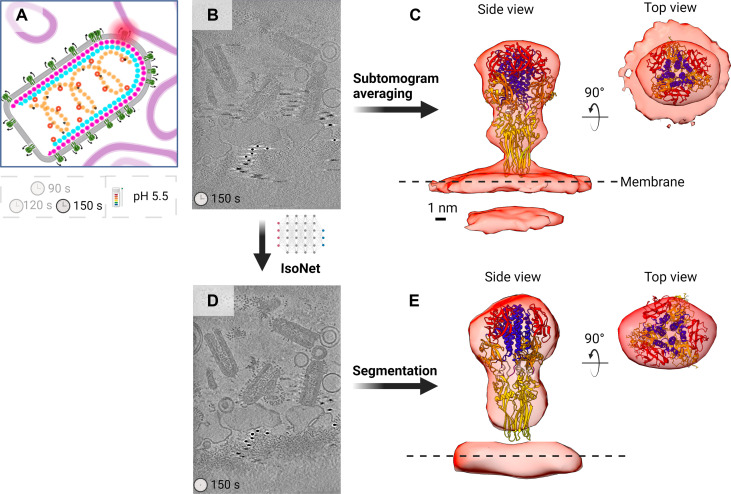
Comparison between a subtomogram average of the postfusion trimer and a subotmogram corresponding to a postfusion trimer from a missing wedge-corrected tomogram. (**A**) Cartoon representation of the experimental condition and schematic suggesting that of the three time points that were imaged (90, 120, and 150 s), only tomograms from the 150-s dataset were used for the averaging and segmentation shown here. (**B**) XY slice view of a raw tomogram from the 150-s low-pH dataset. (**C**) Side view (left) and top view (right) of the crystal structure of the postfusion G ectodomain (PDB ID: 5I2M) (*9*) fitted into a low-resolution density map obtained by subtomogram averaging of Gs from 150-s tomograms. (**D**) Tomogram from (B) after missing wedge correction and deconvolution (*37*). (**E**) Side view (left) and top view (right) of a subtomogram segmented from an IsoNet-corrected tomogram with the postfusion trimer ectodomain (5I2M) fitted into the density. The figure was created in BioRender.com.

Tomograms of VSV virions incubated with liposomes at pH 5.5 for 90, 120, and 150 s (Fig. 4A) exhibited a markedly heterogeneous population of G proteins; some were intermediates en route to postfusion collapse, and most had already reorganized into postfusion trimers. VSV virions are decorated by an average of 1205 G protomers, which amounts to ~400 trimers in the pre- and postfusion configurations (*34*). We generally observed 9 to 15 virions per tomogram in our data and estimated that these contained ~12.4 intermediates per tomogram, across all datasets. This number is an underestimation as it fails to account for intermediates that might have only been observable by examination of XZ or YZ orthogonal planes; nonetheless, it highlights the intermediates’ scarcity and transient nature. Visual assessment of those intermediates, further discussed below, revealed astounding conformational diversity. Any attempt at averaging G proteins captured assuming similar intermediate states was rendered unsuccessful because of their inherent heterogeneity and flexibility.

A subtomogram average was produced in Particle Estimation for Electron Tomography (PEET) using 447 particles manually picked (*35*, *36*) from post–150-s incubation tomograms (Fig. 4B). We strived toward indiscriminate sampling without focusing on G morphologies to minimize unconscious bias. The resulting density corresponded to the postfusion trimer that looks like an inverted cone, ~12 nm in length (Fig. 4C). This further demonstrates how at 150 s of incubation at pH 5.5, this structure dominates the conformational landscape of G proteins represented in our data. Missing wedge correction of a tomogram (*37*) from the same dataset (Fig. 4D) enabled direct segmentation of isotropic postfusion trimer densities (Fig. 4E). The comparison between averaged (Fig. 4C) and segmented volumes (Fig. 4E) underscores the ability of missing wedge-corrected tomograms to accurately reflect the protein morphology. Together, considering these findings, we concluded that (i) direct analysis of individual G proteins may be necessary for structural characterization of intermediates using cryo-ET and that (ii) observation and segmentation from missing wedge corrected tomograms is a well-suited approach.

### Spatiotemporal dynamics of G protein intermediates

**Fig. 5.**
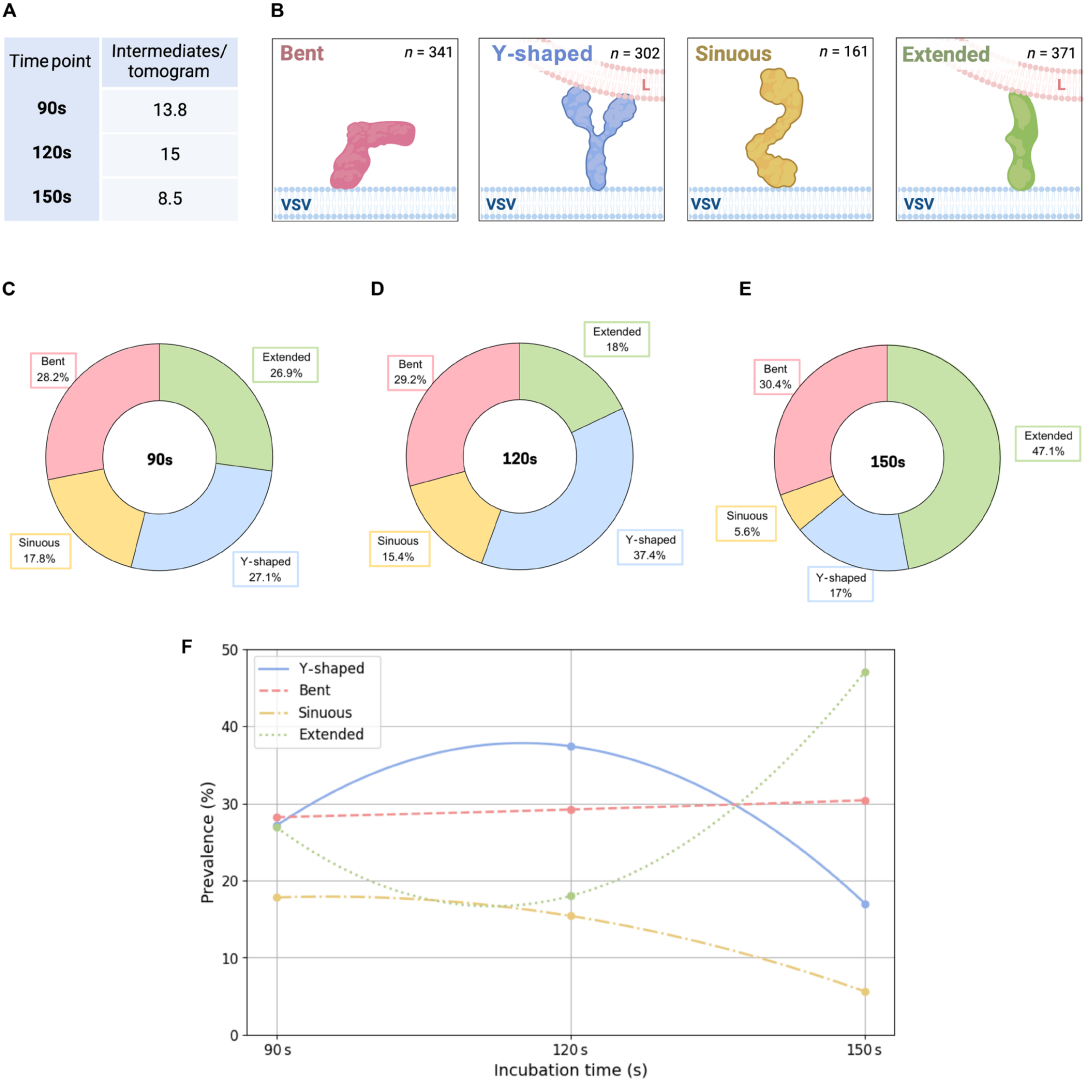
Morphological categorization of observed intermediates and their temporal distribution. (**A**) Table exhibiting the number of intermediates observed on average per tomogram within each dataset (90, 120, and 150 s) (**B**) Cartoon depiction of the four morphological groups: bent, Y-shaped, sinuous, and extended. (**C** to **E**) Pie chart depicting distribution of intermediate groups at each time point. (**F**) Prevalence of intermediate morphological groups plotted against incubation time.

Detecting minority populations of intermediate states is inherently challenging because of their rarity and pleomorphic nature. We set out to apply in situ cryo-ET to effectively capture transient fusion intermediates within low-pH tomograms and analyze them by quantitative methods, visual inspection, and direct segmentation from missing wedge-corrected density maps (*37*). We cataloged a total of 1175 intermediates across 90-, 120-, and 150-s postincubation datasets, which contained 13.8, 15, and 8.5 intermediates per tomogram, respectively (Fig. 5A). Subsequently, these G intermediates were classified into four broad morphological groups (Fig. 5B): (i) bent, (ii) sinuous, (iii) Y-shaped, and (iv) extended intermediates. These groups are reductionist, as G exists at a dynamic conformational continuum between its pre- and postfusion configurations; however, they provided an initial framework for categorizing the general appearance of G intermediates observed in tomograms and gauging their prevalence over time (Fig. 5).

In hindsight, we interpret that bent and sinuous intermediates were largely composed of monomeric G proteins that exhibit conformational flexibilities in the DIV-DIII hinge region, the DII domain, as well as the membrane-proximal C-terminal region. We hypothesize that the Y-shaped morphological group includes two main types of intermediates: highly transient open trimers and seemingly stable parallel dimers. Extended intermediates included both monomers and parallel dimers that uniformly projected their fusion domain(s) vertical to the viral envelope, exceeding ~16 nm in length. Intermediates from each morphological group have occasionally been observed in contact with liposomes.

We assessed the temporal distribution of these intermediate morphologies (Fig. 5, C to F). At the earliest, 90-s time point (Fig. 5C), there is a relatively even distribution of observed intermediates with slight dominance of bent intermediates (28.2%) relative to extended (26.9%), Y-shaped (27.1%), and sinuous intermediates (17.8%). At 120 s (Fig. 5D), there is clear dominance of Y-shaped intermediates (37.4%) followed by bent (29.2%), extended (18.0%), and sinuous intermediates (15.5%). The distribution shifts at 150 s (Fig. 5E) at which extended intermediates make up 47.1% of all intermediates observed, followed by bent (30.4%), Y-shaped (17.0%), and sinuous intermediates (5.6%). It is known that the extension of G protomers is reversible, and protomers are expected to alternate between extended and nonextended conformations (bent and sinuous); this reversibility is likely why we observe the abundance of extended intermediates oscillate from 26.9% at 90 s, to 18.0% at 120 s, to 47.1% at 150 s.

The localization of these intermediates relative to liposome contact zones was also analyzed (fig. S4). At 90s, 84.4% of all intermediates are found distal to a contact zone, notably outnumbering those within contact zone (9.0%) and contact zone-proximal (6.6%) categories (fig. S4C). At 120 s, contact zone (27.6%) and contact zone-proximal (27.6%) intermediates together make up 55.2% of all those recorded, notably outnumbering contact zone-distal intermediates (44.2%) (fig. S4D). At 150 s, this trend continues with a slight decrease in contact zone and contact zone-proximal intermediates; contact zone-distal intermediates dominate (62.6%), followed by contact zone (21.8%) and contact zone-proximal intermediates (15.4%) (fig. S4E). Across all time points, contact zone-distal intermediates are most abundant, but there is a clear trend in the increasing abundance of contact zone and contact zone-proximal intermediates over time (fig. S4B). While the 150-s time point has the lowest abundance of intermediates among low-pH datasets (8.5 intermediates per tomogram; Fig. 5A), this decrease corresponds to a substantial 29.1% increase in extended intermediates (Fig. 5F). These observations highlight that, at endosomal pH, G proteins tend to transition into more extended conformations that are most capable of membrane engagement.

Through quantitative analysis (Fig. 5 and fig. S4), we have identified several trends regarding the spatiotemporal distribution of G protein intermediates: (i) Y-shaped intermediates, which encompass both open trimers and dimeric G proteins, are predominant in the early stages of incubation. (ii) The prevalence of bent intermediates, largely made up of monomers, remains constant over time. (iii) Sinuous intermediates are scarce and predominantly observed during the early stages of incubation. (iv) Extended intermediates become more prevalent over time, consistent with their role in later stages of fusion (i.e., membrane engagement). (v) Contact zone intermediates are most frequent at the 120-s time point. These quantitative insights allowed us to contextualize 3D structural data from low-pH tomograms and reconstruct possible pathways of G-mediated fusion.

### Low pH–driven transition to G trimer open conformation

Upon incubation with liposomes at endosomal pH (i.e., pH = 5.5), G trimers were captured transitioning into an open conformation: a first step in the low pH–induced reconfiguration of G (Fig. 6). This rearrangement begins with refolding of the C-terminal part of the ectodomain, triggered by protonation of specific histidine residues (*19*, *38*, *39*). Such protonation of the C-terminal histidines imparts mobility to the remainder of the G ectodomain, enabling rotation around the DIV-DIII hinge and movement of the fusion domain (DIV). The fusion domains (DIV) of certain open intermediates captured here appear to project beyond 94° (estimated ~97° to 142°; Fig. 6, B and I to K), which is anticipated from DIV-DIII rotation alone. Further vertical projection of the fusion domain (DIV) would require the lengthening of F2, a central helix in the trimerization domain (DII), and recruitment of segment F1, to form a longer helix designated F (*15*). Therefore, we interpret this intermediate to be a transient state bordering on trimer dissociation, likely characterized by simultaneous DIV-DIII hinge rotation and partial F2 helix elongation. Our observed open conformational states are otherwise consistent with those previously envisioned from the known pre- and postfusion states of G trimer ectodomain (*22*, *23*). Furthermore, our analysis of Y-shaped intermediates’ temporal dynamics corroborates the role of open trimers in early stages of fusion (Fig. 5, C to F).

**Fig. 6.**
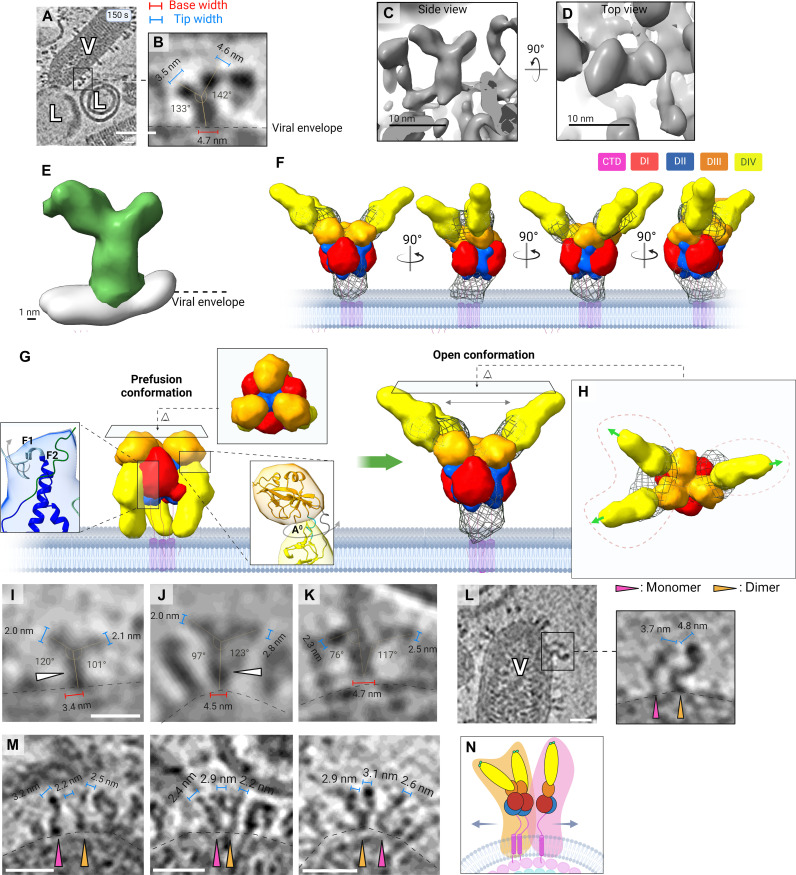
Open conformations of the G trimer and its dissociation into dimers and monomers. (**A** and **B**) Enlarged XY slice views of the tomogram containing density attributed to an open intermediate and pertaining to panels (C) to (H), with annotated dimensions. (**C** and **D**) Side and top views of raw tomographic density from (A) and (B). Scale bar, 10 nm. (**E**) Segmented volume from (C) with local membrane density displayed. (**F**) A tomogram density-guided model of proposed domain localization for the open intermediate, consisting of three G protomers, with domains I to IV encased with 15-Å masks, rotated in 90° increments, and colored as previously assigned in literature (*9*, *15*). Masks were generated by low-pass filtering of known domains, and the mesh volume corresponds to tomographic density the model was guided by. Models in (F) are not to scale. (**G**) Proposed model for trimer opening along with top-down views of both a prefusion trimer (5I2S) and the open-trimer model, which is fitted into the original intermediate density from (E), represented as a mesh. (**H**) Top view of open-intermediate model. Lime green arrows indicate the direction in which the fusion loops of each protomer are pointing. Red dotted lines are drawn around two protomers on the left and one on the right, indicating that their fusion loops appear to be pointed in opposing directions. (**I** to **K**) Enlarged XY slice views of tomograms containing density attributed to open trimers with annotated base/tip widths and DIV projection angle. (**L** and **M**) Enlarged XY slice views of tomograms containing densities attributed to an extended monomer in tandem with a dimer, interpreted in (**N**). Scale bars, 20 nm. G proteins in (L) both exhibit sinuous bending in the membrane-proximal C-terminal region. The figure was created in BioRender.com.

An open intermediate captured after 120 s of incubation (Fig. 6, A to E) was used to produce a model (Fig. 6F) for the ectodomain of this intermediate (residues 1 to 422), guided by tomographic density; this allows us to gauge the relative domain localization within this trimer. Our modeling was informed by prior circular dichroism and small-angle x-ray scattering experiments (*24*). Results from those experiments indicated that (i) ectodomain G undergoes considerable domain shifts in response to pH changes and (ii) these pH-induced movements do not lead to any substantial changes in the secondary structure content. Therefore, we segmented isotropic tomographic density attributed to the open intermediate (Fig. 6, A to E) and used it to guide modeling. Our approach included rigid fitting of domains I to IV into the segmented density and refinement of flexible hinges, as further detailed in Methods. From inspection of tomographic density and the resulting model, it appears that two of the protomers are “opening” with their fusion domains (DIV) closer to one another, while the third protomer has its fusion loops pointed in the opposite direction (Fig. 6, G and H). This raises the possibility of dimer formation during or after low pH–triggered trimer disassembly as a step in the transitional pathway. Dimers and monomers are frequently observed in tandem, lending credence to this possibility (Fig. 6, L to N); however, static snapshots acquired using cryo-ET do not provide us with reliable insight into the mechanics of this process. Our observations of dimeric as well as monomeric intermediates are further detailed below. While parallel dimers of G have never been observed or discussed before, monomers of ectodomain G have been subject to previous research (*23*, *24*, *26*).

### Conformational diversity of G monomers

Monomers of G were captured adopting a diverse array of configurations emerging from three main sources of variability: (i) the degree of DIV-DIII hinge rotation, (ii) the level of F1 helix elongation, and (iii) presence or absence of a bending moment in the membrane-proximal C-terminal region. The degree of rotation at the DIV-DIII hinge and projection relative to the viral envelope (Fig. 7, A to L) predisposed monomers to either the bent (~50° to 130° projection) or the extended intermediate (~130° to 150° projection) morphological category (Fig. 5B). However, as mentioned before, these categories are reductionist; close examination of individual monomers reveals vast conformational diversity even within morphological categories independently of liposome contact, which is highlighted in Fig. 7. Plot profiles for the G monomer tip width have an average of 2.8 ± 0.37 nm (*n* = 15).

**Fig. 6. Fig. 7.**
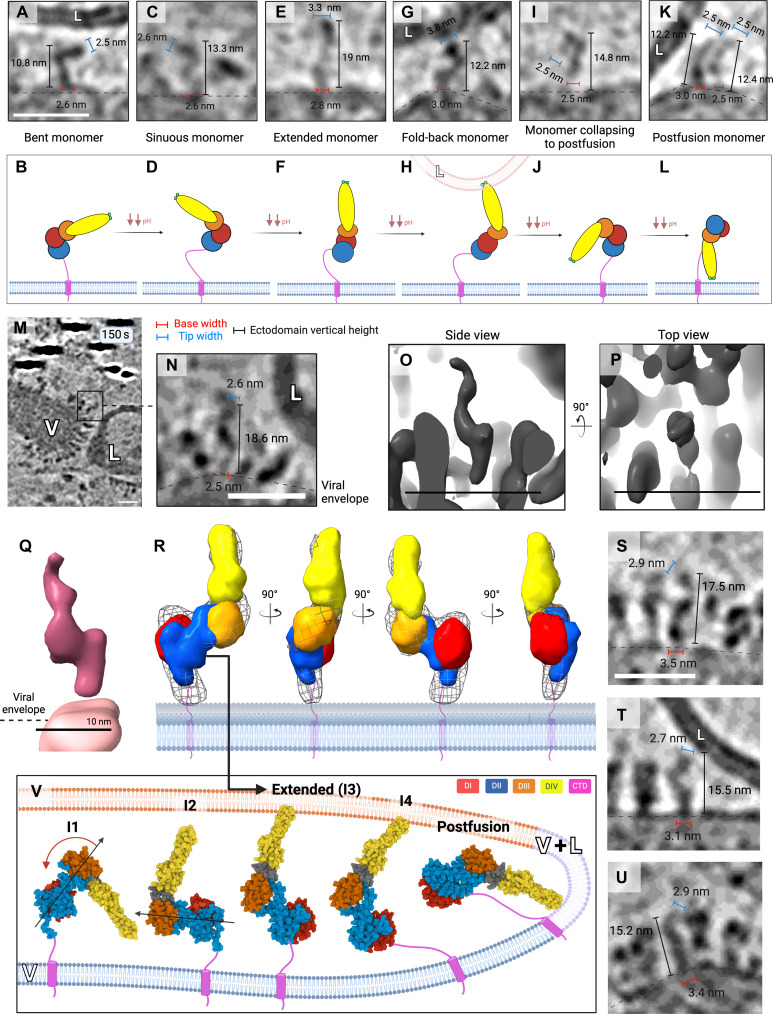
Visualization and modeling of G monomer conformational states. (**A** to **L**) Enlarged XY slice views of monomeric intermediates in various conformations (top row), interpreted below (second row). Scale bar, 20 nm. (**M** to **R**) Modeling of extended monomer domain localization guided by tomographic density, produced as described in Fig. 6, A to F. (**S** to **U**) XY tomogram slices showing additional examples of extended monomeric intermediates, with height and width annotated as in previous panels. (**V**) Morphing model (*40*) for the pre- to postfusion structural transition of G monomers. I1 and I2 were obtained by morphing the prefusion protomer (PDB ID: 5I2S) with the putative extended intermediate (I3). I3 was morphed with the postfusion protomer, yielding I4. Intermediates are not to scale. Black arrows across I1 and I2 signify the plane defined by domains DII and DIII. The red arrow signifies ~120° anticlockwise rotation of the DII-DIII plane in the transition from I1 to I2. All conformations from (V) are visualized using Mol* Viewer (*62*). The figure was created in BioRender.com.

Bent monomeric intermediates (*n* = 341; Fig. 7, A and B) are likely among the first intermediates to emerge on the pre- to postfusion transitional pathway, resulting from trimer disassembly. Low pH favors projection of G fusion loops vertical to the viral envelope (*24*). This step is likely to involve refolding within the membrane-proximal C-terminal region in the ectodomain that gives rise to monomers exhibiting sinuous morphology (Fig. 5B and fig. S5), as elaborated further below (Fig. 7, C, D, and V). However, bent intermediates persistently make up 28.2 to 30.4% of all intermediates across all time points (Fig. 5, C to F). Their consistent prevalence implies that bent intermediates might be regenerated by reconfiguration of later-stage intermediates, such as extended or fold-back intermediates that are dissociated from the target membrane, as in previous reports that corroborate the reversibility of G protomer extension (*23*).

**Fig. 8.**
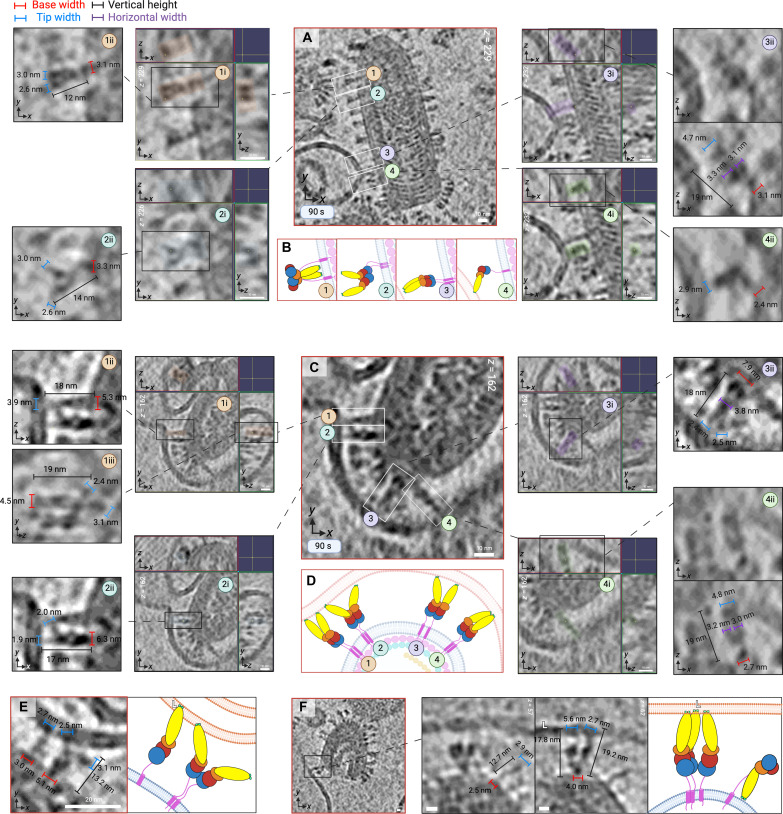
Extended intermediates of G engaging the target membrane. (**A**) Enlarged XY slice view of a tomogram with four sites of interest encased in numerically annotated white rectangles (1 to 4). Each has two subsequent zoomed insets, i and ii; insets 1i to 4i show three orthogonal views (XY, YZ, and ZX) of each intermediate, while insets 1ii to 4ii show a view from one of those orthogonal planes, annotated with dimensions. All intermediates from (A) are hence interpreted in (**B**). (**C**) The central panel is an XY slice view of a tomogram which has four points of interest annotated (1 to 4), followed by zoomed insets of orthogonal views (XY, YZ, and ZX) for each intermediate (1i to 4i). Same as in (A), dimensions are annotated in subsequent insets (1ii, 1iii, and 2ii to 4ii). (C) is interpreted in (**D**). (**E**) Enlarged XY slice view of an extended monomer in tandem with a Y-shaped extended dimer that has one of its fusion loops inserted into the liposomal membrane, alongside a cartoon interpretation (right) of the scene captured in the tomogram. (**F**) Enlarged XY slice views of a DIV-DIV-superimposing extended dimer and an extended monomer in tandem, both engaging a liposomal membrane. To their right is a postfusion monomer. Zoomed inset depicts intermediates with the viral membrane aligned horizontally; two XY slices are depicted for clarity (left and middle), with annotated dimensions for the postfusion (left) and extended (middle) intermediates. The leftmost panel is a cartoon interpretation of the scene. The figure was created in BioRender.com.

**Fig. 9.**
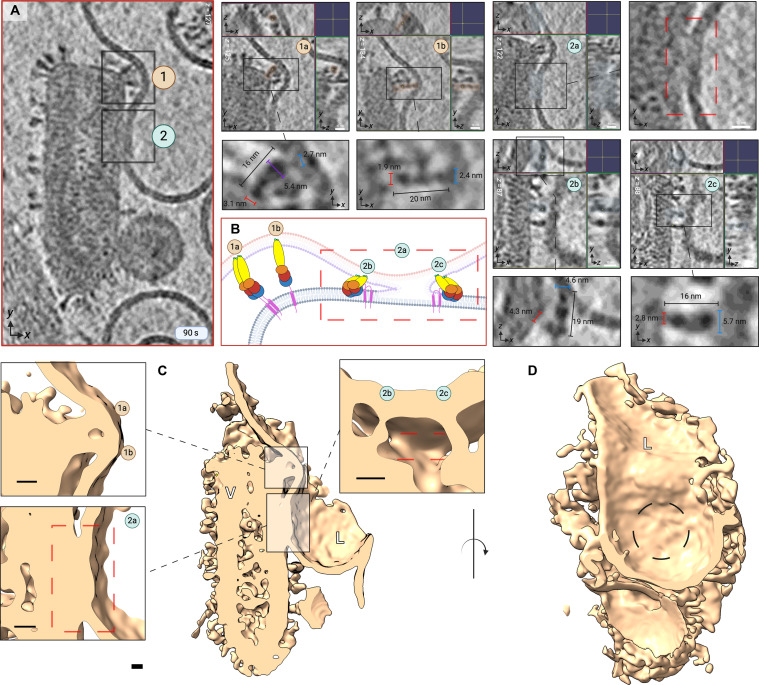
Hemifusion mediated by fold-back of G extended intermediates. (**A**) Enlarged XY slice views of a tomogram from the 90-s time point with two sites containing intermediates numerically annotated (1 and 2) and encased in black boxes. Site 1 contains two intermediates (1a and 1b). Site 2 contains two intermediates (2b and 2c) that are clustered around a ~50-nm hemifusion diaphragm annotated as 2a and highlighted within a dashed red rectangle. Panels containing three orthogonal views (XY, YZ, and ZX) are displayed for all intermediates from sites 1 and 2 (1a, 1b, 2b, and 2c), alongside close-up views of their morphology with dimensions annotated in blue (tip width) and red (base width). (**B**) Cartoon interpretation of intermediates displayed in (A). (**C**) A side cross view of the 3D rendering of the hemifusion site from (A) is shown. Zoomed insets display side views of extended intermediates 1a and 2b (upper left), the hemifusion diaphragm encased in a red dashed rectangle (site 2a, lower left) and extended intermediates clustering around the diaphragm, 2b and 2c (upper right). (**D**) Density from (C) rotated by 90°. The circular surface of the hemifusion diaphragm can be seen (black dotted circle). Scale bar, 10 nm. The figure was created in BioRender.com.

Complete projection of the fusion loops is characteristic of extended intermediates (Figs. 5B and 7, E and F), and this renders them capable of interacting with a target membrane. Our interpretation is that extended monomers captured here are generally characterized by lengthened trimerization domain F helices, a 94° rotation around the DIV-DIII hinge, and the fusion domain projected at the top of the trimerization domain. Extended monomers were observed distal to the target membrane (Fig. 7, E, F, S, and U) as well as at the contact zone, or penetrating the target membrane (Fig. 7T; see Figs. 8A and 9A). Upon membrane engagement, monomers were captured folding back to generate apposition of the viral and target membranes (Fig. 7, G and H). Once detached from the target membrane, fold-back monomers collapse, with their fusion loops directed toward the viral envelope (Fig. 7, I and J), en route to a postfusion conformation (Fig. 7, K and L). Membrane penetration and hemifusion events resulted from cooperative fold-back of monomeric and dimeric extended intermediates were also visualized and presented previously (Fig. 2) and as follows (see below).

We set out to gauge conformational changes in the pre- to extended to postfusion transition, while using an intermediate directly observed in tomograms. A representative extended monomer was identified (Fig. 7, M and N) and evaluated in 3D to establish isotropic resolution (Fig. 7, O and P). A model for the intermediate was produced to gauge domain localization, guided by the tomographic density (Fig. 7, Q and R) as previously in Fig. 6. Subsequently, a multidimensional scaling method (*40*) was used to compute putative intermediate structures between (i) the prefusion G protomer [Protein Data Bank (PDB) ID: 5I2S] and our extended intermediate model and (ii) our extended intermediate model and the postfusion G protomer (PDB ID: 5I2M). Four intermediates computed by morphing are displayed as conformations I1, I2, and I4 in Fig. 7V, where our extended intermediate is represented as I3. A ~120° counterclockwise rotation of the DII-DIII block appears to take place during the transition from putative intermediate I1 to I2 (Fig. 7V), possibly driven by bending in the membrane-proximal C-terminal region. In our cryo–electron tomograms, we did observe a subpopulation of G monomers (*n* = 161) that exhibited notable bending in this membrane-proximal region (Figs. 6I and 7C and fig. S5), resembling the ectodomain structure of I2 (Fig. 7V). These intermediates were allocated to the sinuous morphological category, of which the prevalence decays from 17.8 to 5.6% over time (Figs. 5, B and F, and 7, C and D). Quantitative analysis further highlights the likely involvement of sinuous intermediates in early stages of the fusion process (Fig. 5, C to F). Taking these observations altogether, we conclude that a large conformational spectrum is explored by G monomers via rotation around the DIV-DIII hinge, DII F2 helix elongation, and refolding of the seemingly crucial (*16*, *18*–*20*) but structurally uncharacterized membrane-proximal and transmembrane C-terminal regions.

### Hemifusion driven by extended dimeric and monomeric intermediates

Vertical projection of the G fusion domain serves to expose its bipartite fusion loops to target membranes, enabling membrane penetration and G-mediated fusion by extended intermediates. This step is immediately observable after 90 s of incubation at pH 5.5. Extended intermediates (~17 to 19 nm) were captured distal and proximal to membrane contact zones, as well as inserted into liposomes. Figures 8 and 9 show that intermediates were inspected from all three orthogonal views (XY, YZ, and ZX) and their oligomeric state was deduced by comparison of their tip width(s) to that of an average monomer (2.8 ± 0.37 nm). Our observations suggest that extended intermediates (*n* = 371) include both monomeric and dimeric G proteins capable of target membrane engagement and apposition (Figs. 7 to 9).

Two main configurations of G parallel dimers were captured, all uniformly characterized by association via the membrane-proximal C-terminal region. The first kind are Y-shaped dimers (*n* < 302; Fig. 5B), characterized by continuous parallel adhesion up to the fusion domains and separation of the fusion domains with a degree of flexibility at the DIV-DIII hinge (Fig. 8A, panels 2, 2i, and 2ii interpreted as Fig. 8B, panel 2). Y-shaped dimers are capable of engaging target membranes via one (Fig. 8C, panels 2, 2i, and 2ii interpreted in Fig. 8D; Fig. 8E) or both fusion loops (Fig. 8C, panels 4, 4i, and 4ii interpreted in Fig. 8D; Fig. 8F). G protomers constituting Y-shaped dimers were confirmed to have an average of 2.6 ± 0.30 nm in width (*n* = 16), largely consistent with the previous assessment of G monomer monomers’ tip width (2.8 ± 0.37 nm). The second kind of parallel dimer are extended dimers whose lateral interface encompasses the fusion domains; as expected, these intermediates were found to have tip widths of ~4.5 to 5.6 nm owing to DIV-DIV superimposition (Fig. 8A, panels 3i and 3ii interpreted in Fig. 8B, panel 3; Fig. 8C, panels 1, 1i to 1iii, 4, 4i and 4ii, interpreted in Fig. 8D; and Fig. 9A, panels 1a, 2b, and 2c). The assembly of DIV-DIV superimposing extended G dimers is therefore likely to contribute to the increased population of extended intermediates engaging target membranes at 150 s (Fig. 5, E and F).

As shown in Figs. 8 and 9, we capture monomers, “Y-shaped” dimers and extended dimers penetrating membranes followed by their fold-back and juxtaposition of apposing membranes, resulting in hemifusion and mixing of proximal leaflets. Surrounding the interface between the opposing membranes, G dimers and monomers are both observed facilitating the formation of a ~50-nm hemifusion diaphragm. In addition, monomeric and dimeric G intermediates were captured assuming postfusion configurations, both distal and proximal to VSV-liposome contact zones (Fig. 8A, panels 1, 1i, and 1ii; Fig. 8F), indicating that extended intermediates collapse into a postfusion conformation before reassembling into trimers.

We suggest that G dimers exist at a conformational continuum betwesen the Y-shaped and extended morphology. At the 150-s time point, the prevalence of Y-shaped intermediates declines by 20.4% (Fig. 5E), simultaneously as in extended intermediates become more prevalent by 29.1% (Fig. 5E); these trends are consistent with the possibility of Y-shaped dimers transitioning into an extended morphology over the course of exposure to endosomal pH, as previously reported for monomers (*24*). Furthermore, it lends credence to a mechanism that entails fusion domain convergence at the target membrane, as extended dimers were only observed at VSV-liposome contact zones.

## DISCUSSION

**Fig. 10.**
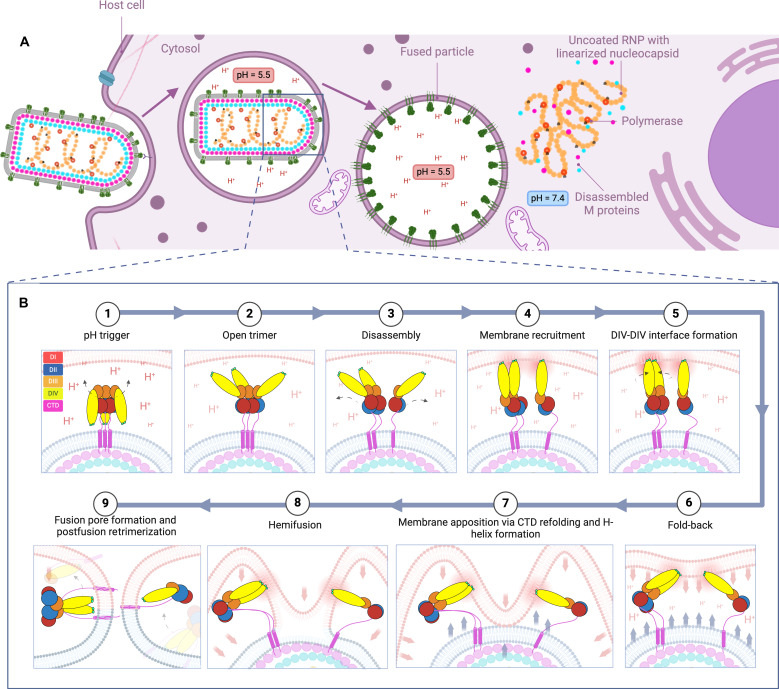
Proposed mechanism of VSV G-mediated fusion in vivo. (**A**) Cartoon illustrating a model for VSV host cell entry including RNP uncoating and linearization. (**B**) Model of interaction between G protein and the endosomal membrane upon acidification to pH 5.5. The figure was created in BioRender.com.

Enveloped viruses are decorated with viral fusion proteins which mediate the fusion process by lowering associated kinetic barriers; this is achieved using the free energy released during their structural transition (*1*). VSV G is a prototype for class III Gs (Fig. 1C) which also include HSV-1 gB, EBV gB, and baculovirus gp64 (*6*). In this study, we incubated VSV virions with liposomes at endosomal pH (i.e., pH = 5.5) and imaged them at different time points (90, 120, and 150 s) using cryo-ET (fig. S1) to capture (i) the conformational intermediates of G protein (Figs. 5 to 9) and (ii) membrane remodeling intermediates that occur during the class III viral fusion process (Figs. 2, 3, and 9). Integrated structural and quantitative analyses of these conformational intermediates have allowed us to propose a sequential mechanism of pH-triggered class III viral membrane fusion through nine conformational steps, as illustrated in Fig. 10.

First, the G trimer assumes a stable prefusion conformation (“1”) at neutral pH, which gets destabilized in the event of endosomal acidification. G trimers then enter a transient open conformation (“2”) (Fig. 6) due to pH-induced refolding of the C-terminal region of the ectodomain and rotation of flexible DIV-DIII hinges (Fig. 6G). Subsequent recruitment of F1 segment residues to lengthen the F2 central helix in the trimerization domain leads to F helix generation; this process results in repulsion at the trimer interface and causes it to disassemble (Fig. 6G).

Upon their disassembly, open trimers (“3”) act as precursors to intermediates of lower oligomeric states, including parallel dimers and monomers (“4” and “5”). Monomers of G explore a conformational continuum via rotation at the DIV-DIII hinge, refolding of the DII helix F1, and sinuous bending in the membrane-proximal C-terminal region, or the G stem. G dimers were captured assuming (i) a variable Y-shaped morphology, characterized by fusion loops in separated protomers and some degree of flexibility around the DIV-DIII hinge (Fig. 8A, panels 2, 2i, and 2i interpreted as Fig. 8B, panel 2), and (ii) a fully extended conformation, characterized by continuous lateral adhesion of protomers, and formation of a DIV-DIV interface (Fig. 8A, panels 3i and 3ii interpreted as Fig. 8B, panel 3; Fig. 8C, panels 1, 1i to 1iii, 4, 4i, and 4ii; Figs. 9A and 7B, panels 1a, 2b, and 2c). 

Both groups of dimers are uniformly characterized by association via the G stem, and possibly the TMD; this may explain why previous biochemical studies failed to detect G dimers at acidic pH using native mass spectrometry, as they used a form of G ectodomain generated by limited proteolysis that lacks the membrane-proximal region (*24*). Both monomers and dimers of G can extend and insert their fusion loops into the target membrane.

Target membrane engagement is followed by folding back of extended dimeric and monomeric intermediates (“6”, “7” and “8”). Fold-back applies the external pressure that is needed to overcome the hydration barrier to fusion and allow for mixing of the proximal leaflets’ hydrophobic tails and formation of a hemifusion stalk (Fig. 2D). The hemifusion stalk expands, forming a wider hemifusion diaphragm (Fig. 2E). The accompanying conformational change within G most likely involves refolding within the G stem as well as formation of the C-terminal helix H.

Additional mixing of the apposing bilayers’ distal leaflets gives rise to a fusion pore (Fig. 2F) through which contents of either membranes become exposed to one another. At the end of the fusion process, G dimers and monomers (“9″) collapse to a postfusion conformation and reform trimers. Through analysis of fused particles (Fig. 3) as well as DIPs (fig. S2) and uncoated RNP helices (fig. S3), we propose that the VSV nucleocapsid may disassemble into free matrix proteins and a linearized, polymerase-bond RNP genome after the fusion process (Fig. 10).

Cryo-ET has allowed us to visualize membrane fusion events and transient conformational intermediates of VSV G with unprecedented clarity, shedding light on the mechanism of class III fusion catalysis and postfusion alterations in VSV’s molecular architecture. This work demonstrates the strengths of cryo-ET in characterizing dynamic biological processes, but it also highlights the method’s inherent limitations and needed advances in the field. Detailed structural analysis of transient intermediate states at high resolution necessitates averaging hundreds of thousands of homogeneous particles of each unique conformation; the structural heterogeneity of intermediates captured here poses a challenge. We partially overcome these obstacles by missing wedge correction and denoising of our tomograms (*37*), which enabled isotropic resolution, direct visualization, and characterization of molecular features.

Because of the need to freeze samples, cryo-ET can only capture snapshots of dynamic conformations, and hence, it lacks continuous temporal resolution. Mechanistic interpretation and reconstruction of the chronological sequence is left for interpretation by the researcher. We attempted to address this limitation by collecting tilt series at three discrete time points (90, 120, and 150 s) and relying on laborious manual quantification to achieve a degree of time resolution. In future studies, the current approach might greatly benefit from minimizing the human factor and using machine learning to identify and assess intermediate states of surface Gs within tomograms as objectively and efficiently as possible.

Visual inspection of extended dimers and Y-shaped dimers, as well as their abundance, makes for a compelling argument that these intermediates are being formed. We calculated the average tip width of monomers to be ~2.8 nm; this value was used to inform our identification of Y-shaped dimers by having two such tips. Accordingly, extend dimers had tip widths around ~4.5 to 5.6 nm owing to DIV-DIV superimposition. VSV G has a relatively small bipartate fusion loop, comprising only four hydrophobic residues per protomer, so it may be favorable for fusion to have multiple tightly apposed fusion loops engaging the target membrane. However, it is difficult to gauge the relative stabilities of monomers and dimers using cryo-ET; the energetics underlying their formation and fusogenic activity would be an interesting subject to investigate in future studies using alternative methods.

Furthermore, follow-up cryo-ET studies would benefit from investigating alternative experimental conditions, such as varying time points, pH levels, and liposome compositions. To delineate early G conformational intermediate states and fusion intermediates with greater specificity, exploring earlier (e.g., 30 to 60 s) and later incubation time points (e.g., 10 to 30 min) might be advantageous, respectively. Although this study primarily focused on modeling VSV escape from the late endosome at pH 5.5, VSV G-mediated fusion is known to occur over a wider pH range (pH 6.2 to 5.0). A comparative analysis of the abundance of specific intermediates at these different pH values across equivalent time points could yield insights into the differential escape mechanisms of VSV from various endosomal compartments. Maintaining the virions at a slightly higher pH (e.g., pH 5.7 to 6.2) may facilitate more transitions between prefusion to postfusion and post- to prefusion configurations, potentially reducing the number of actual fusion events. Future research should also consider the specific protein and lipid composition of endosomes containing internalized VSV virions to refine liposome-based models that more accurately replicate the physiological sites of VSV fusion and uncoating within endosomes.

This research showcases a prospective and enticing way of capturing transient intermediate states that are essential for mechanistic interpretation of dynamic biological processes. Several exciting works have used similar methods to studying viral entry and fusion in recent years (*41*–*43*). The integration of focused-ion-beam milling for cryo-ET, along with advancements in correlative light and electron microscopy, signals a promising future for biophysical studies of viral entry mechanisms. We anticipate that in the coming years, these techniques will enhance our collective understanding of viral infections, hopefully leading to breakthroughs in their prevention and treatment.

## METHODS

### Virus isolation

Isolation of VSV virions (Indiana serotype, San Juan strain) followed a protocol previously described (*44*). Full viral particles with prefusion G trimers decorated on the viral envelope were isolated from media as previously reported (*21*). In brief, the inoculum was plaque purified, following several passages in HeLa cells that have a minimal multiplicity of infection; this prevents the formation of truncated DIPs. The medium stock was subjected to centrifugation with 20% sucrose cushion at 30,000*g* for 2.5 hours and resuspended in phosphate-buffered saline (PBS, pH 7.4). Large aggregates were then removed by a low-speed centrifugation at 12,000*g* for 5 min. The resulting suspension was subsequently pelleted at 100,000*g* for 10 min. The pellet was resuspended in a small volume of PBS and directly used for the incubation with liposomes and cryo-ET sample preparation.

### Liposome preparation

Preparation of liposomes followed a protocol previously described (*29*). Phospholipids, consisting of DOPC, DOPS, and cholesterol in a molar ratio of 4:2:1 were dissolved and mixed in chloroform at a concentration of 25 mg/ml. The chloroform was then evaporated under a nitrogen gas flow and the phospholipids were further dried to form a film. The resulting lipid film was suspended in 250 μl of liposome buffer [10 mM Hepes, 100 mM NaCl, and 50 mM sodium citrate (pH 7.5)], vortexed, frozen in liquid nitrogen, thawed, and vortexed again. The freeze-thaw-vortex steps were repeated five times. The mixture was extruded 21 times through a 0.2-μm polycarbonate membrane filter by a Mini Extruder (Avanti Polar Lipids) to obtain a transparent solution containing small vesicles with a diameter of 100 to 200 nm. The liposomes were kept at 4°C for further use.

### Cryo-ET sample preparation

To prepare cryo-ET grids at low-pH condition, liposomes were titrated to pH 5.5 with citrate buffer. Approximately 2.5 μl of the purified VSV virions in PBS were pipetted onto glow-discharged, thin continuous carbon film–coated lacey grids (Ted Pella) and incubated for 1 min at room temperature. Excess liquid containing virions was manually blotted away after with filter paper from the edge of the grid. Acidified liposomes (3 μl) mixed with 10-nm gold beads as fiducial markers were applied to the grids. After a 90-, 120-, or 150-s incubation period, the grids were blotted again (blot force 16, blotting time 20s) using a Vitrobot (Mark IV; Thermo Fisher Scientific Co.).

### Cryo-ET imaging and drift correction

Grids were loaded into a Titan Krios 300-kV transmission electron microscope (Thermo Fisher Scientific Co.) equipped with a Gatan imaging filter (GIF) Quantum LS and a Gatan K3 detector for tomography. The beam was aligned in nanoprobe mode, and the GIF slit width was set to 20 eV. Tomographic tilt series were collected between −60° and 60° with a 3° angular increment in dose-symmetry scheme (*45*) by SerialEM 3.8.0 (*46*) at a nominal magnification of ×64,000 (calibrated pixel size 0.68 Å, super mode). The total dose of each tilt series was ~120e^−^/Å^2^, constantly distributed over the tilt series. Tilt series movies were recorded in dose-fraction mode with a defocus range 2.5 to 4.5 μm. Frames in each movie were then motion corrected and binned two times with the graphics processing unit–accelerated program MotionCor2 (*47*) to generate a micrograph in a tilt series.

### Cryo-ET data processing and segmentation

A total of 100 tilt series across three postincubation time points were collected and reconstructed following the standard protocol provided by IMOD (*35*). Reconstructed tomograms binned to a pixel size of 8.16 Å were corrected for the missing wedge artifact and deconvoluted with IsoNet (*37*) using defocus values estimated with CTFFIND4 (*48*); while IsoNet does minimize the missing wedge, slight missing wedge artifacts may still persist after correction, and vary across different tomograms. Segmentation was performed using ChimeraX (*49*) after visual inspection to ascertain isotropic resolution of intermediates.

To identify fused particles for segmentation, we established a criterion for distinguishing fused particles from potential DIPs during visual inspection of tomograms. Previous research has reported that VSV DIPs differ from standard virus particles only by (i) the presence of shorter helical nucleocapsids that display a reduced number of striations compared to the larger, standard virus particles or (ii) breakage of the untruncated helical nucleocapsid into two closely apposed segments tightly encased by the viral envelope (*50*). Fused particles were differentiated from DIPs on the basis of the premise that DIPs are not characterized by RNP linearization.

### Subtomogram averaging

Four hundred forty-seven Gs were manually selected from three IsoNet-corrected 150-s postincubation tomograms (pixel size = 8.16 Å) in 3dmod. Tomograms were visualized suing 3dmod. G proteins were identified as densities projecting up to ~18 to 20 nm away from the viral envelope. A total of 447 G proteins were manually selected from two tomograms, each defined by two points: one at the base nearest to the membrane and another at the peak, farthest from the membrane. These coordinates were saved as .mod files, and StalkInit was used to generate particle orientations in PEET as previously described (*51*). Particle coordinates selected using missing wedge-corrected and deconvolved tomograms (pixel size = 8.16 Å) were therefore applied onto corresponding unbinned tomograms (pixel size = 1.36 Å), and a subtomogram average was produced using the PEET package (*52*, *53*). The TomoNet software package (*36*) was used to streamline the subtomogram averaging workflow.

### Modeling G protein conformational intermediates

Domains were rigidly docked into tomogram densities by allowing motion in the flexible hinge regions. The ISOLDE tool (*54*) in ChimeraX was then used to restrain all secondary structure elements, selectively release hinges and simulate them to reach the most stable conformation. Masks (15 Å) were generated around domains as modeled to depict their proposed localization.

### Negative-stain imaging of VSV RNP

The nucleocapsid and the RNP were purified from virions (*55*–*57*). The virions were incubated with a lysis buffer [20 mM tris-HCl (pH 8.0 at 25°C), 400 mM NaCl, 1.9% (w/v) Triton X-100, 1 mM 1,4-dithiothreitol, 5% (v/v) glycerol] (*57*). The sample was layered on top of a 20% (v/v) glycerol TE [20 mM tris-HCl (pH 8.0) and 1 mM EDTA) cushion and centrifuged at 150,000*g* for 4 hours at 4°C. The pellet containing the RNP was resuspended in TE containing 10% (v/v) glycerol. For negative-stain EM, 3 μl of the purified VSV RNP chain was applied to glow-discharged carbon-coated 300-mesh Cu EM grid and then stained with 2% (w/v) uranyl acetate. Negative-stain EM images were collected using an FEI Tecnai T20 transmission electron microscope equipped with a charge-coupled device camera.

### Cryo-EM imaging of VSV RNP

For the cryo-EM sample, 3 μl of the purified VSV RNP was applied to glow-discharged lacey carbon grids with a supporting ultrathin carbon film (Ted Pella). The grids were then blotted with filter paper and flash frozen in liquid ethane using an FEI Vitrobot Mark IV. Cryo-EM images were collected using a Titan Krios electron microscope equipped with a Gatan K3 camera. 2D classification result was obtained with particles from 500 images using cryoSPARC (box size = 440 Å).
