# Supplementary material for: Capturing intermediates and membrane remodeling in class III viral fusion
Source: Sci Adv. 2024 Dec 4;10(49):eadn8579. doi: 10.1126/sciadv.adn8579 (PMC11616707; doi:10.1126/sciadv.adn8579)
Supplement: Supplementary file 1 — Figs. S1 to S5 [file sciadv.adn8579_sm.pdf]

Science Advances
AAAS

Supplementary Materials for

Capturing intermediates and membrane remodeling in class III viral fusion

Lenka Milojević *et al.*

Corresponding author: Z. Hong Zhou, hong.zhou@ucla.edu

Sci. Adv. **10**, eadn8579 (2024)
DOI: 10.1126/sciadv.adn8579

This PDF file includes:

Figs. S1 to S5

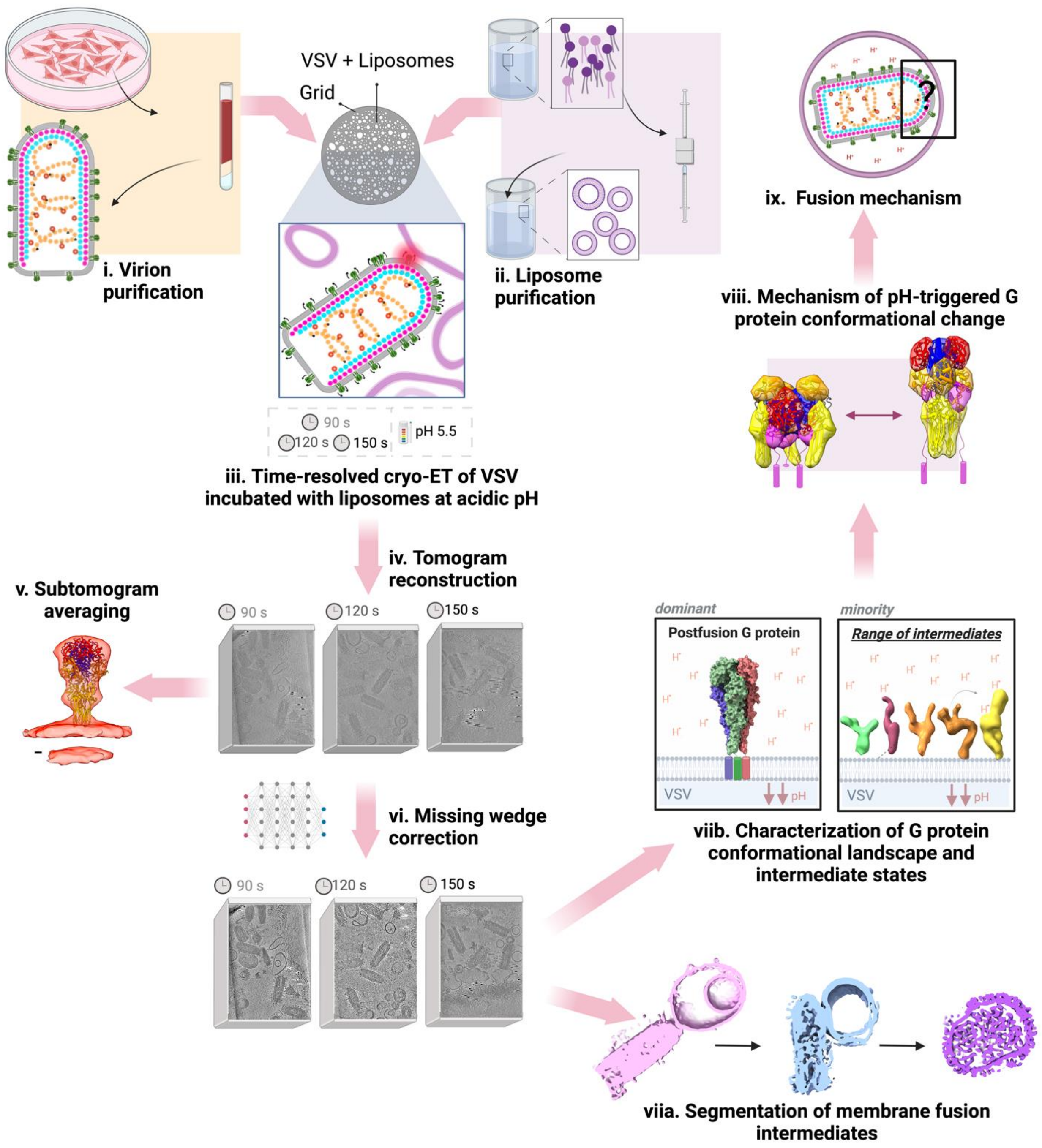

Fig. S1. Visual summary of methods and workflow. VSV virions purified from HeLa cells (i) and late endosome-mimicking liposomes (ii) were incubated on cryoEM grids for 90s, 120s and 150s in a pH 5.5 citric acid buffer (iii) prior to flash-freezing and tilt series collection. Following tomogram reconstruction (iv), a subtomogram average of VSV G was produced, reflecting the dominant postfusion conformation (v). Raw tomograms (iv) were then subjected to missing wedge-correction (vi) and used for characterization of membrane fusion intermediates (vii, a) and the G conformational landscape (vii, b). Resulting observations were used to produce the proposed mechanism of G-mediated class III fusion catalysis (viii-ix).

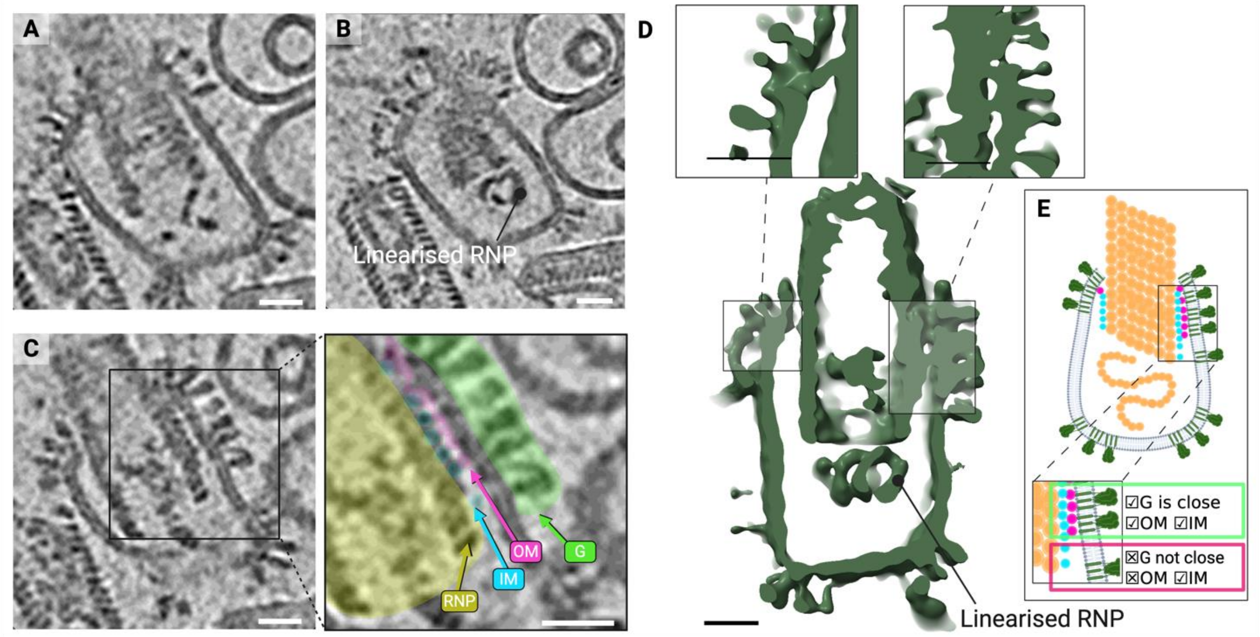

Fig. S2. Uncoated helical RNP breaking out of its envelope.

(**A**) Enlarged XY slice view of a tomogram containing the RNP helix breaking out of its envelope. (**B**) A different slice of the same feature from (A) to show linearized RNP. (**C**) Another XY slice view of the same feature (left) with a zoomed inset (right) showing the layering within the distressed nucleocapsid. OM proteins are only observed underneath G proteins, likely binding electrostatically to its endodomain. (**D**) 3D rendering of the feature from (A-C) with zoomed insets highlighting additional densities corresponding to OM proteins underneath G proteins. Inspection of the large cross-view reveals increased thickness of the capsid in the G-proximal region, attributed to additional presence of OM. (**E**) Cartoon representation of feature displayed in (A-D).

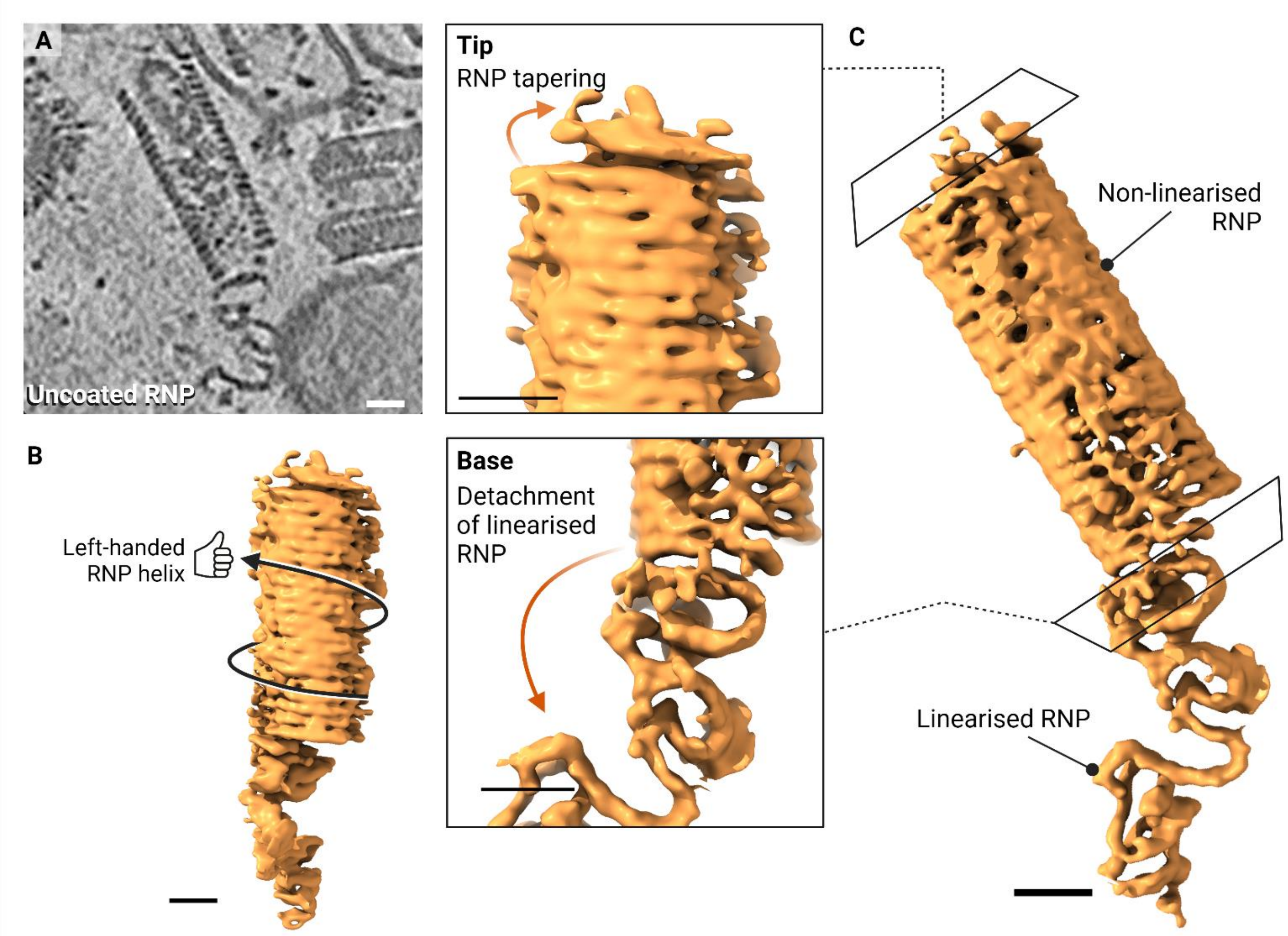

Fig. S3. Uncoated nucleocapsid linearizing at the ends.

(**A**) Enlarged XY slice view of a tomogram containing an uncoated nucleocapsid sequestering VSV's non-segmented negative strand RNA genome. (**B**) 3D rendering of nucleocapsid from (A) with the RNP helix vertically aligned to discern left-handed twist. (**C**) An enlarged view of the 3D rendering from (B) displayed at the same orientation as in the tomogram (A). Zoomed insets of the top (tip region) and bottom (base region) of the capsid show different degrees of linearization; one turn of the RNP helix appears to be tapering off at the tip, while a significant portion of the base has already linearized. Tip and base identification within the nucleocapsid were determined by assessment of the twist.

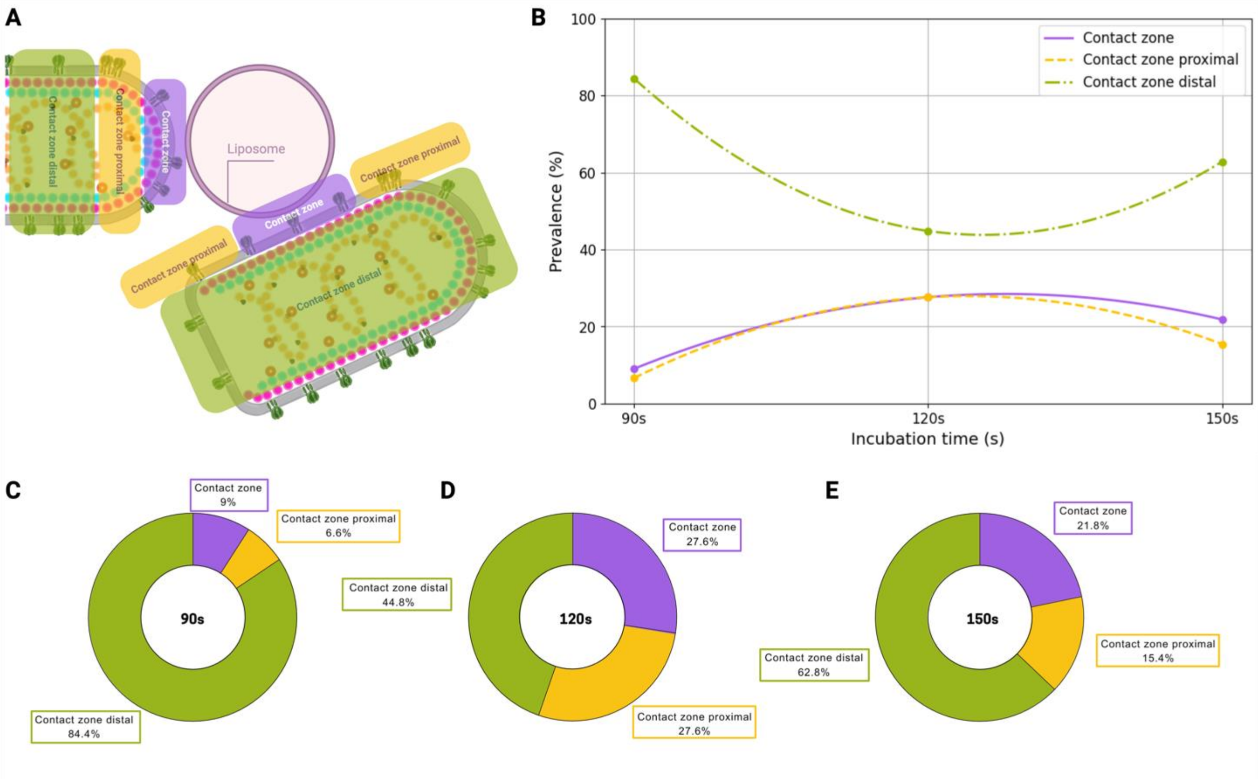

Fig. S4. Localization of G intermediates relative to liposome contact zones over time.

(**A**) Cartoon schematic depicting and defining localization groups as contact zone intermediates, contact zone-proximal intermediates and contact zone-distal intermediates. (**B**) Line graph showing the prevalence of localization groups across incubation timepoints. (**C-E**) Pie charts representing data from (B) including exact percentages.

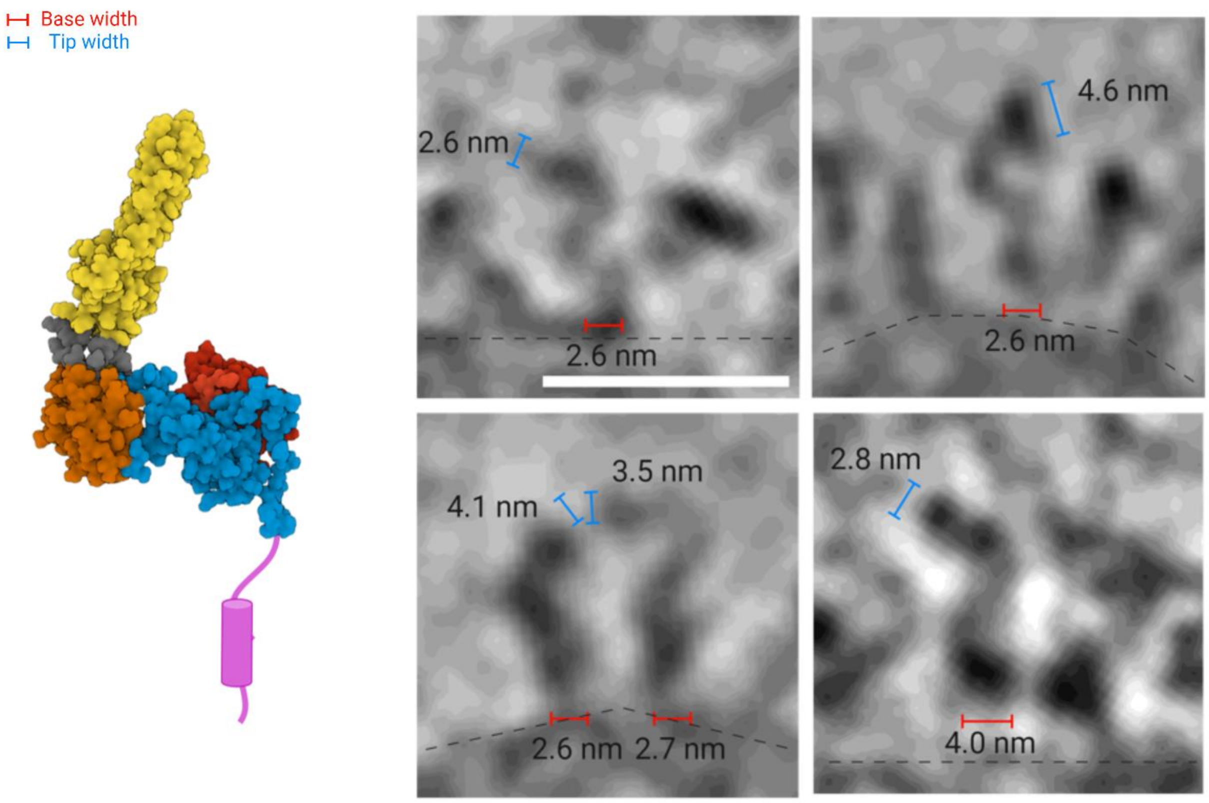

Fig. S5. Sinuous intermediates.

Left: Intermediate I2 from Fig. 7V, obtained by morphing between the prefusion G protomer (5I2S) and the extended intermediate modeled in Fig. 7, M to R. Right: Representative examples of sinuous intermediates observed in tomograms. Tip width annotated in blue, base width annotated in red. Membrane annotated with dashed black line. Scalebar is 20 nm.
